# Olfactory dysfunction in the scenario of COVID-19 pandemic in patients screened by the telemonitoring

**DOI:** 10.31744/einstein_journal/2021AO6204

**Published:** 2021-09-30

**Authors:** Raíssa Camelo Valletta, Leandro Azevedo de Camargo, Stela Oliveira Rodrigues, Sarah Vidal da Silva, Mateus Capuzzo Gonçalves, Nathálya Rodrigues Queiroz, Arlindo Rodrigues Galvão, Melissa Ameloti Gomes Avelino

**Affiliations:** 1 Universidade Federal de Goiás GoiâniaGO Brazil Universidade Federal de Goiás, Goiânia, GO, Brazil.; 2 Hospital das Clínicas Universidade Federal de Goiás GoiâniaGO Brazil Hospital das Clínicas, Universidade Federal de Goiás, Goiânia, GO, Brazil.; 3 Pontifícia Universidade Católica de Goiás GoiâniaGO Brazil Pontifícia Universidade Católica de Goiás, Goiânia, GO, Brazil.

**Keywords:** Olfaction disorders, COVID-19, Coronavirus infections, Taste disorders, Population health, Epidemiology, Telemonitoring

## Abstract

**Objective:**

To assess the clinical and epidemiological profile of patients with olfactory dysfunction in the scenario of COVID-19 pandemic.

**Methods:**

The study selected patients with loss of smell, previously screened by telemonitoring system of the Municipal Health Department of Goiânia (GO), Brazil, who agreed to answer a questionnaire about COVID-19 symptoms and findings of exams. The interviews were conducted by six otolaryngologists, who applied the specific questionnaire, over the phone.

**Results:**

A total of 13,910 patients underwent telemonitoring, and 627 (4.51%) had olfactory loss complaints. Out of them, 330 were included in the survey. We observed a higher prevalence of altered smell in women (67%), and in patients aged under 50 years (86%). In most cases the manifestations had a sudden onset (70%), and in the first 5 days of illness (80%). The most prevalent associated symptom was a change in taste (89%), and only 2.7% of interviewed patients required hospitalization.

**Conclusion:**

Anosmia in COVID-19 is more prevalent in females and individuals aged under 50 years. It is a relevant initial symptom predictive of the disease, together with dysgeusia.

## INTRODUCTION

In December 2019, a new coronavirus started circulating in China, in the city of Wuhan, province of Hubei, causing a disease that is currently known as coronavirus disease 2019 (COVID-19), which accounts for the current pandemic. The virus, due to its genomic similarities with the severe acute respiratory syndrome virus (SARS-CoV), which caused the severe acute respiratory syndrome (SARS) epidemic, in China, in 2002, was named SARS-CoV-2. It is a single-stranded, enveloped RNA virus of the *Coronaviridae* family, which shares similarities with the SARS-CoV-type viruses of the same family, which are bat-borne. However, the original host of the SARS-CoV-2 is still unknown.^( 1 , 2 )^

Clinical evidence supports that viral transmission takes place through droplets, fomites and aerosol particles, with indication of low risk vertical transmission.^( 3 )^ The incubation period is estimated at 5 days, and the disease has different clinical presentations, varying from cold-like symptoms to septic shock.^( 3 , 4 )^ The most common symptoms are fever, dry cough, dyspnea, chest pain, myalgia and fatigue. However, other symptoms may be observed, such as headache, abdominal pain, diarrhea, nausea, vomiting, anosmia and ageusia.^( 1 )^ Olfactory disorders have been a frequent complaint (up to 70%) which, sometimes, may occur isolated from common respiratory and systemic symptoms of COVID-19, with a higher incidence in females.^( 2 , 5 )^

The risk factors for progression to severe disease identified to date include advanced age (age over 60 years) and presence of comorbidities (such as *diabetes mellitus* , cardiovascular diseases, hypertension and pulmonary diseases).^( 1 , 3 , 4 )^ Some studies suggest that anosmia is commonly associated with mild to moderate disease, and usually occurs in the early course of symptoms.^( 6 )^

The diagnosis of the disease is based on clinical suspicion combined with laboratory tests. Reverse transcription polymerase chain reaction (RT-PCR) has a provenly higher sensitivity rate (approximately 80%), when performed three or more days after onset of symptoms. It is performed on respiratory samples (such as nasopharyngeal) and considered the gold standard for diagnosis.^( 3 )^

Olfactory changes in the context of viral respiratory infections are common and, overall, they occur in association with rhinorrhea and nasal congestion. However, in SARS-CoV-2 infection, olfactory disorders, in addition to being more frequent than those associated with other viruses, may occur in isolation, as an early symptom, during or after resolution of other nasal symptoms. Sudden-onset loss of smell, in the context of the current pandemic, is highly suggestive of infection by SARS-CoV-2, and it is advisable to immediately isolate the patient presenting this symptom. Since anosmia usually occurs in the early stage of the disease, the onset of this symptom as a positive predictor of COVID-19 has a major role in preventing virus propagation.^( 2 , 6 )^

The mechanisms involved in olfactory changes (which can manifest as hyposmia or anosmia) are not yet clear.^( 5 )^ There is also significant coexistence between olfactory and gustatory dysfunctions, either due to distinct changes caused by the virus in gustatory tissue, or due to loss of the gustatory function present in retronasal olfaction.^( 6 )^

## OBJECTIVE

To assess the clinical and epidemiological profile of patients with olfactory dysfunction in the context of the COVID-19 pandemic.

## METHODS

A descriptive, epidemiological study investigating the population with COVID-19 in the state of Goiás, (GO), Brazil via telemonitoring, conducted by a team of otolaryngologists. This study enrolled only patients referring loss of smell, who were in quarantine at the time of the interview, either for suggestive symptoms or laboratory-confirmed diagnosis, and screened through general COVID-19 telemonitoring performed by the Municipal Health Department of Goiânia, jointly with the *Universidade Federal de Goiás* (UFG). The study was reviewed and approved by the local Ethics Committee, and is registered in *Plataforma Brasil* under CAAE: 35260820.8.0000.5078 and opinion 4.199.318.

Interviews were conducted by the staff on a daily basis, between June 4 and July 14, 2020. Verbal consent was obtained for application of a questionnaire ( Appendix 1 ) addressing epidemiological data, such as sex, age and occupation, as well as clinical data such as initial symptom, associated symptoms, duration of olfactory changes, day and form of onset, comorbidities, need for hospitalization and treatment received.

The data obtained was input on a worksheet in Google Drive, openly accessible to the group of researchers. These results are presented in a descriptive fashion, showing the prevalence of these events in the study population.

## RESULTS

Between June 4 and July 14, 2020, a total of 13,910 patients with a clinical or laboratory-confirmed diagnosis of COVID-19 were telemonitored by the Municipal Health Department and UFG, of whom 627 referred altered smell, resulting in a 4.51% prevalence of olfactory changes in this population.

**Figure 1:**
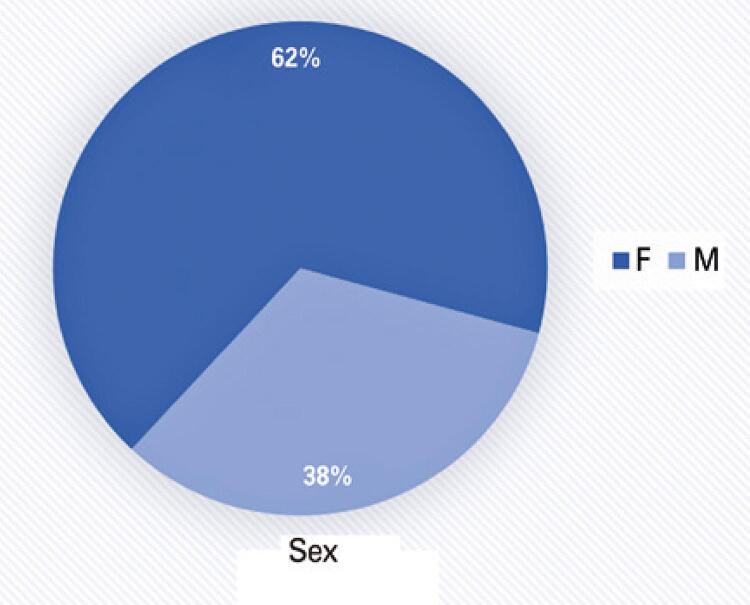
Distribution by sex

Of the 627 patients with olfactory changes who were screened, 62% were females and 38% were males. A total of 330 patients were contacted and enrolled after agreeing to answer a specific questionnaire. Of these, 62% were females ( Figure 1 ). In respect to age, 86% of patients were aged under 50 years; of these, 36% were aged under 30 years. As for the professional occupation, approximately 23% of respondents were healthcare professionals. Interviews were conducted, on average, within the first 10 days after disease onset, and in 64.2% of patients the diagnosis had already been confirmed by specific tests.

F: female; M: male.

**Figure 2:**
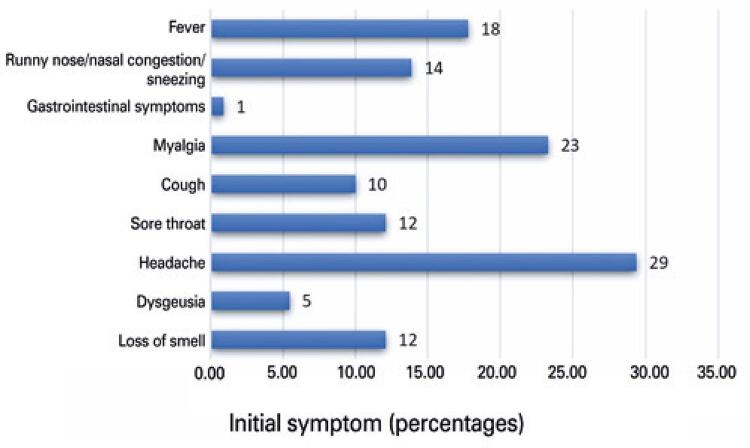
Inicial symptom

**Figure 3:**
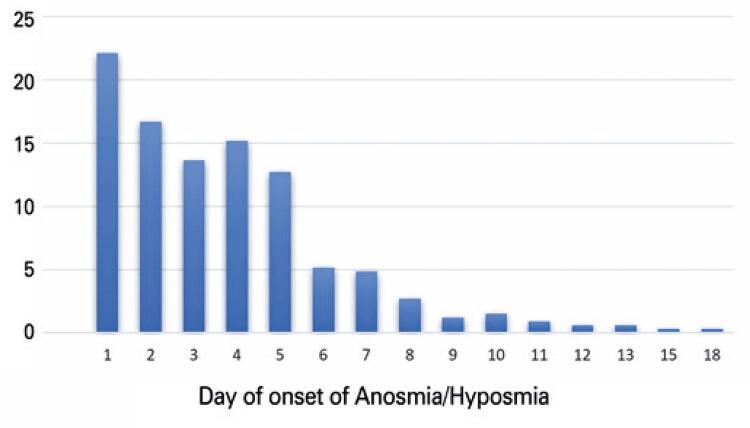
Day of onset of loss of smell

As for the clinical picture, the most prevalent initial symptom was headache in nearly 30% of patients, followed by myalgia (23%) and fever (18%); loss of smell was the initial symptom in 12% of patients ( Figure 2 ). A subjective quantification of the loss of smell showed 81.3% reported total loss of smell (anosmia), whereas 18.6% reported partial loss of smell (hyposmia). The onset of olfactory loss took place in the first 5 days for 80% of patients, whereas most of them reported sudden onset (70%) ( Figure 3 ). Total recovery of smell within 10 days after disease onset was observed in 13% of patients, 38% had partial improvement, and 48% had persistent anosmia at the time of the interview. Patients who did not fully recover their olfaction continued to be followed up by telemonitoring by the otolaryngology team, with calls after 15 and 30 days until resolution of anosmia.

**Figure 4:**
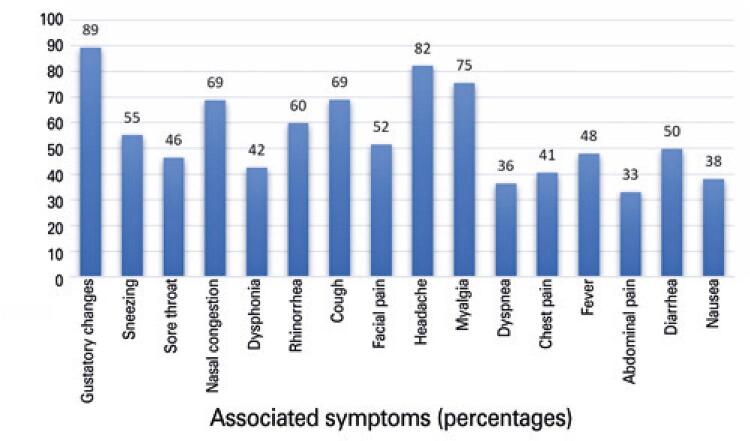
Associated symptoms

When reviewing the associated symptoms ( Figure 4 ), dysgeusia stood out for being present in 89% of patients, pointing to a very strong relation with anosmia/hyposmia. Headache and myalgia were the second and third most prevalent, occurring in 82% and 75% of patients, respectively. Nasal congestion was reported by 69% of patients, rhinorrhea by 60%, cough by 69%, dysphonia by 42%, and dyspnea by 36%. The prevalence of obesity was 20%, of which 14% had grade 1 obesity, 37% were overweight, and 39% had a normal body mass index.

In respect to the treatment received, 50% received regular analgesics, 40% received azithromycin, 25% ivermectin, 12% corticosteroids, and 5.5% received hydroxychloroquine. Treatment was received in an outpatient setting in 97.3% of patients, and only 2.7% of patients required hospital admission, of which 77% were hospitalized for less than 5 days. Of the 330 patients, only 0.6% required admission to an intensive care unit (ICU).

In terms of the ear-nose-throat (ENT) history, approximately 20% of patients had a nasal disorder, such as rhinitis (10.3%), rhinosinusitis (7.8%) and nasal congestion (2.1%). Other comorbidities were also investigated, and 9.7% reported hypertension, 5.1% diabetes, and 6.9% asthma; no patients reported heart failure.

As for additional tests, 83% of patients did not undergo any lab test. The most frequent test was complete blood count, performed in 17% of patients, with normal results in 73% of cases, leukopenia in 7%, and thrombocytopenia in 17%. D-dimer was performed in 1.9% of patients and results were normal in all of them. A chest X-ray was performed in 5.8% of patients, and 0.9% were abnormal; however, 14.7% of patients underwent a chest computed tomography; 47% of tests were normal, 30% had less than 25% lung involvement, 12% had less than 50% involvement, and only 2% had more than 50% lung involvement suggestive of viral pneumonia.

## DISCUSSION

According to the current scientific literature, the prevalence of SARS-CoV-2-related anosmia is very variable (5% to 85.6%). This probably happens due to the different methodologies used, and the enrollment of suspected patients.^( 1 , 6 )^ This study found a rate of 4.51% among patients telemonitored by the Municipal Health Department and UFG, which is a low number, probably due to the presence of many suspected patients without laboratory confirmation, leading to a selection bias. When reviewing the data of patients selected for the questionnaire (n=330), a higher concordance with the literature was found, probably because all these patients were referring loss of smell, and since this symptom seems to be a predictor of the disease, the selection led to a lower number of suspected cases that were truly negative.

Recent studies performed on population-based samples in periods preceding the current COVID-19 pandemic identified as risk factors for olfactory disorders, male sex and advanced age, with statistically significant differences between sexes and an age range between 18 and 50 years (women had better olfactory function based on subjective tests in this age range).^( 7 , 8 )^ On the other hand, descriptive studies performed in COVID-19 populations have shown that olfactory changes are predominant in the female sex; however, the mean age of the patients assessed was similar.^( 2 , 9 - 12 )^ In this study, similarities were found with previous studies, with 67.2% of females and a mean age of 37 years.

Regarding the proportion of patients with a diagnosis confirmed by specific COVID-19 tests in this sample, 64.2% had laboratory confirmation at the time of the interview, and all others had a suggestive clinical picture, but with no laboratory confirmation. The assessment of anosmia and hyposmia in these two groups is in line with a large, multicenter study by Parma et al., in which the two study groups showed no statistically different prevalence of olfactory disorders (only a higher-intensity loss of smell in the laboratory-confirmed group of patients).^( 13 )^The same assessment was conducted with the data in this study, validating patients with a laboratory-confirmed diagnosis had a higher rate of anosmia (82%), and those with a clinical diagnosis had a higher rate of hyposmia (72%). Nevertheless, the rate of ageusia in both groups was high and similar (85% in the first group and 93% in the second group). This analysis suggests that, although approximately 36% of patient cohort assessed did not have a confirmed COVID-19 diagnosis, the clinical presentation in all respondents indicates a high equivalence and reliability of the data obtained.

In respect to the intensity of olfactory disorders referred in this sample (81.3% anosmia and 18.6% hyposmia), similar data were found in a study with a similar methodology (online survey), with a prevalence of 81.7% and 76.1% for anosmia, and 15.1% and 17.9% of hyposmia, in patients in quarantine or with a laboratory-confirmed diagnosis, respectively.^( 10 )^ Hopkins et al., and Kosugi et al., found a similar pattern of distribution for partial or total loss of smell.^( 9 , 14 )^

Olfactory disorder was the first symptom of COVID-19 in 11.8% of cases in Lechien et al., similar to the 12% found in this study; however the rate of presentation as a first symptom varies greatly in different case series (3% to 83%).^( 2 , 11 , 14 - 22 )^In this case series, loss of smell had sudden onset in 70% of cases, and occurred within the first five days of disease in 80% of respondents, similarly to the findings of Hopkins et al., and Klopfenstein et al.,^( 9 , 12 )^

As for progression, 48% of patients had persistent olfactory changes for up to 10^th^ days after symptom onset. Despite the persistent complaint, the pathogenesis of olfactory disorders in COVID-19 involves non-neuronal mechanisms, which suggests that permanent total loss of smell is unlikely.^( 23 )^

In respect to the association of olfactory and gustatory disorders, other similar studies support these findings (89%), with rates ranging between 85% to 90.65%.^( 2 , 9 , 12 )^The presence of concomitant nasal symptoms in this sample was approximately 20%, lower than found in other studies.^( 10 , 14 )^ The presence of unspecific symptoms in this series, such as headache (in 85% of patients) and myalgia (in 75%), was similar to the findings of a study that objectively investigated olfactory changes in patients with a laboratory-confirmed diagnosis.^( 24 )^

In this analysis, patients referred a longer time for olfaction improvement, even for partial improvement, in comparison with that of other studies.^( 10 )^However, the time frames are similar to those in another Brazilian series, which also found a longer recovery time in patients with olfactory dysfunction related to COVID-19, in comparison with other viral infections.^( 14 )^

Cases requiring hospital admission or intensive care were less frequent than those reported by Klopfenstein et al., in a retrospective study of patients seeking medical attention who had a confirmed COVID-19 diagnosis.^( 12 )^ This could be explained by the fact that this study was performed through a phone survey and, therefore, in patients that, at first, had mild to moderate disease. However, the role of anosmia as a potential protective factor against severe disease cannot be ignored, as already hypothesized in other studies.^( 2 )^ In fact, there is limited data on anosmia in patients with moderate to severe disease, maybe because the focus in these cases is on treatment or investigation of other potentially more severe factors, such as dyspnea. Intensive care physicians and patients with severe disease may not realize or may underestimate the relevance of anosmia as a symptom amidst a severe systemic clinical presentation.

This study has several limitations such as data collection from subjective subject evaluations and is, therefore, subject to cultural and measurement biases. However, a comprehensive sample was obtained from one single region, and it was performed through direct contact between otolaryngologists and patients, which allows for detailed characterization of the olfactory disorder landscape during the increased number of cases in Goiás.

Another limitation is the absence of laboratory confirmation of all cases; however, it is worth reinforcing the presence of anosmia as an isolated symptom has a positive association with a COVID-19 diagnosis when compared with other respiratory viruses, and this association is even stronger upon absence of other nasal symptoms.^( 16 , 21 )^

Also, laboratory tests have different capacities for detection of SARS-CoV-2, depending on the day of collection, the technique and the type of material collected for the sample, where nasopharyngeal and oropharyngeal swabs (most commonly used RT-PCR sample collection techniques) provide only moderate detection capacity, with a considerable rate of false-negative results.^( 25 - 29 )^ This corroborates the importance of olfactory changes in presuming a COVID-19 diagnosis.

Sudden hyposmia or anosmia and their associations with changes in the sense of taste and absence of other nasal symptoms (such as nasal congestion and rhinorrhea) must be regarded as a relevant positive predictive factor for a COVID-19 diagnosis, since they tend to appear early on and act as an important tool to curb virus propagation. In the context of the current pandemic, it is agreed that the presence of this clinical picture should raise immediate suspicion of the disease, and it is advisable to put these patients in quarantine, even before confirmatory tests.^( 30 - 32 )^ This association has a high social value, since, in this country, it is still not possible to test all suspected cases. Moreover, since not much is known about this disease, the data in this study can likely contribute to the national and global literature on the subject. It is also worth noting that a suspicion of false-negative results must be raised when the typical clinical presentation is present – which is important considering the low sensitivity of molecular and serological tests in the acute phase.^( 16 , 21 , 30 )^

Regarding the evolution of anosmia, it is advisable to follow up these patients and their evolution, considering that these symptoms lead to significant psychosocial impact.^( 6 )^More studies are needed on the long-term evolution of anosmia and treatment-related factors. A recent study by Brann et al., suggested the involvement of non-neuronal cells as the cause of anosmia in COVID-19, and full recovery is likely in the majority of affected patients.^( 23 )^ Patients in this sample with persistent loss of smell must continue to be followed up on a monthly basis, until complete resolution.

Despite the association between loss of smell and mild COVID-19, there are currently no studies in the literature about this symptom in moderate and severe cases, and this relation may be a measurement bias.^( 33 )^ Therefore, the scientific community must conduct further research on this subject.

## CONCLUSION

In this study, performed via a phone survey with patients with olfactory disorders during quarantine for suspected or laboratory-confirmed COVID-19, the incidence of loss of smell was higher in females, and sudden onset was the most common form. The most common symptom associated with loss of smell was loss of taste, and the presence of both complaints should raise suspicion of COVID-19 diagnosis.

## Appendix 1

**Table:** Appendix 1. Questionnaire epidemiological data

The questionnaire was performed via phone call and paid for by each investigator. After a personal introduction by the interviewer and informing about the telemonitoring project, verbal consent was obtained from the patient to being asked the following questions:
- Sex.
- Age, weight, and height.
- Birth date.
- Occupation.
- What was the first symptom?
- How many days since the onset of the first symptom?
- When and which confirmatory test for COVID-19 was performed?
- What was the day of the change in smell?
- Specify between hyposmia or anosmia.
- Did you undergo any olfactory testing?
- How was the onset of the loss of smell (sudden/progressive)?
- What was the duration of the loss of smell?
- Has the loss of smell improved or it still persists?
- Presence of symptoms: taste dysfunction, sneezing, sore throat, nasal congestion, rhinorrhea, dysphonia, cough, facial pain, headache, myalgia, dyspnea, chest pain, fever, abdominal pain, diarrhea and nausea?
- Which treatment are you receiving?
- Did you require hospitalization?
- Specific history of ENT disorders and/or procedures.
- Presence and past history of comorbidities: diabetes, hypertension, lung disease, diseases with immunological involvement, heart failure, heart diseases other than heart failure, cancer, and other diseases not previously specified.
- Results of ancillary testes: laboratory tests and chest computed tomography.
- Observation for other details not addressed in the previous questions.
Patients with no full recovery from anosmia/hyposmia will be contacted again, 15 and 30 days after the first phone call, and questioned about improvement of olfactory and gustatory symptoms. After 30 days, if the loss of smell persists, the patient is invited for a face-to-face outpatient evaluation and olfactory test.

ENT: ear-nose-throat; COVID-19: coronavirus disease 2019.
