# Supplementary material for: Olfactory dysfunction in the scenario of COVID-19 pandemic in patients screened by the telemonitoring
Source: Einstein (Sao Paulo). 2021 Sep 30;19:eAO6204. doi: 10.31744/einstein_journal/2021AO6204 (PMC8483635; doi:10.31744/einstein_journal/2021AO6204)
Supplement: Translation

# Translation: Distúrbio do olfato no cenário da pandemia de COVID-19 em pacientes triados por telemonitoramento

**Authors:** Raíssa Camelo Valletta, Leandro Azevedo de Camargo, Stela Oliveira Rodrigues, Sarah Vidal da Silva, Mateus Capuzzo Gonçalves, Nathálya Rodrigues Queiroz, Arlindo Rodrigues Galvão, Melissa Ameloti Gomes Avelino

**Affiliations:** 1 Brasil Universidade Federal de Goiás, Goiânia, GO, Brasil.; 2 Brasil Hospital das Clínicas, Universidade Federal de Goiás, Goiânia, GO, Brasil.; 3 Brasil Pontifícia Universidade Católica de Goiás, Goiânia, GO, Brasil.

**Keywords:** Transtornos do olfato, COVID-19, Infecções por coronavírus, Distúrbios do paladar, Saúde da população, Epidemiologia, Telemonitoramento

## Abstract

**Objetivo:**

Avaliar o perfil clínico-epidemiológico dos pacientes com distúrbios olfatórios no cenário de pandemia da COVID-19.

**Métodos:**

Foram selecionados pacientes com queixa de perda de olfato, previamente triados pelo sistema de telemonitoramento da Secretaria Municipal de Saúde de Goiânia (GO), Brasil, que concordaram em responder um questionário sobre sintomas e achados de exame em relação à COVID-19. As entrevistas foram realizadas por seis médicos otorrinolaringologistas que, via telefonema, submeteram os participantes a questionário específico.

**Resultados:**

Dos 13.910 pacientes em telemonitoramento, 627 (4,51%) apresentavam queixas de alteração do olfato, e, destes, 330 foram incluídos na pesquisa. A maior prevalência de alteração do olfato se deu em mulheres (67%), e em pacientes com menos de 50 anos (86%). Na maioria dos casos, as manifestações ocorreram de forma súbita (70%), e nos primeiros 5 dias de doença (80%). O sintoma associado mais prevalente foi alteração do paladar (89%), e houve necessidade de internação hospitalar somente em 2,7% dos pacientes entrevistados.

**Conclusão:**

A anosmia na COVID-19 é mais prevalente no sexo feminino e em pacientes com menos de 50 anos. Juntamente da disgeusia, trata-se de importante sintoma inicial preditivo da doença.

## INTRODUÇÃO

Em dezembro de 2019, um novo coronavírus passou a circular na China, na cidade de Wuhan, província de Hubei, causando a doença atualmente conhecida como doença pelo coronavírus 2019 (COVID-19) e sendo o responsável por essa pandemia atual. O vírus, por sua semelhança genômica com a síndrome respiratória aguda grave (SARS-CoV), que levou à epidemia de síndrome respiratória aguda grave (SRAG) na China, em 2002, foi nomeado SARS-CoV-2. Trata-se de um RNA vírus de fita simples, envelopado, da família *Coronaviridae* , que possui semelhanças com o vírus da mesma família, coronavírus tipo SARS-CoV derivados de morcegos. Contudo, ainda se desconhece o hospedeiro original do SARS-CoV-2.^( 1 , 2 )^

Evidências clínicas corroboram que a transmissão viral ocorre via gotículas, contato com fômites e aerossol, com indícios de baixo risco de transmissão vertical.^( 3 )^O período de incubação é estimado em torno de 5 dias, e a doença possui uma diversidade de apresentações clínicas, as quais variam desde sintomas semelhantes ao resfriado comum até choque séptico.^( 3 , 4 )^Os sintomas mais comumente apresentados são febre, tosse seca, dispneia, dor torácica, mialgia e fadiga. Contudo, demais sintomas podem ser observados, como cefaleia, dor abdominal, diarreia, náuseas, vômitos, anosmia e ageusia.^( 1 )^A queixa de distúrbios olfatórios tem se mostrado frequente (até cerca de 70%) e, por vezes, pode acontecer de maneira isolada dos sintomas respiratórios e sistêmicos comuns da COVID-19, sendo maior sua incidência em indivíduos do sexo feminino.^( 2 , 5 )^

Dentre os fatores de risco para evolução mais grave da doença identificados até o momento estão idosos (maiores de 60 anos) e presença de comorbidades (como *diabetes mellitus* , doenças cardiovasculares, hipertensão arterial e doenças pulmonares).^( 1 , 3 , 4 )^Alguns estudos apontam que a anosmia costuma ter relação com doença de apresentação leve a moderada, geralmente ocorrendo de maneira precoce no curso dos sintomas.^( 6 )^

O diagnóstico da doença baseia-se na suspeição clínica associada a métodos laboratoriais. O exame de transcrição reversa seguida de reação em cadeia da polimerase (RT-PCR) mostrou maior sensibilidade (cerca de 80%), quando realizado a partir do terceiro dia do início dos sintomas. É realizado a partir de amostras respiratórias (como da região da nasofaringe) e considerado o padrão-ouro para diagnóstico.^( 3 )^

As alterações olfatórias no contexto de infecções respiratórias virais são comuns e, em geral, ocorrem associadas com rinorreia e obstrução nasal. Contudo, na infecção pelo SARS-CoV-2, as disfunções olfatórias, além de serem mais frequentes do que as associadas a outros vírus respiratórios, podem surgir isoladamente, como sintoma inicial, durante ou após a resolução dos demais sintomas nasais. A perda de olfato de início súbito, no contexto de pandemia que vivemos atualmente, é altamente sugestiva de infecção pelo SARS-CoV-2, sendo recomendado ao paciente isolamento imediato na presença desse sintoma. Como a anosmia costuma acontecer no início da doença, o estabelecimento desse sintoma como preditor positivo de COVID-19 tem importante papel na prevenção da disseminação do vírus.^( 2 , 6 )^

Os mecanismos implicados nas alterações olfatórias (que podem ser manifestadas como hiposmia ou anosmia) ainda não estão elucidados.^( 5 )^Há também significativa coexistência entre as disfunções olfatórias e gustativas, seja por alterações distintas do vírus no tecido gustativo, ou devido à perda da função gustatória presente na porção olfatória retronasal.^( 6 )^

## OBJETIVO

Avaliar o perfil clínico-epidemiológico dos pacientes com distúrbios olfatórios no cenário de pandemia da COVID-19.

## MÉTODOS

Trata-se de um estudo descritivo epidemiológico no qual foi estudada a população com COVID-19 de Goiás (GO), Brasil via telemonitoramento, realizado por uma equipe de otorrinolaringologistas. Foram entrevistados somente pacientes com queixa de perda de olfato e que estavam cumprindo quarentena no momento da entrevista, seja por quadros clínicos sugestivos ou por confirmação laboratorial, triados a partir do telemonitoramento geral de COVID-19 realizado pela Secretaria de Saúde Municipal de Goiânia em conjunto com Universidade Federal de Goiás (UFG). A pesquisa foi submetida e aprovada pelo Comitê de Ética local, e se encontra cadastrada na Plataforma Brasil com o número CAAE: 35260820.8.0000.5078 e parecer: 4.199.318.

As entrevistas foram realizadas pela equipe diariamente no período entre 4 de junho e 14 de julho de 2020. Foi obtido um consentimento verbal para a aplicação de um questionário ( Apêndice 1 ) abordando dados epidemiológicos, como sexo, idade e profissão, além de dados clínicos, como sintoma inicial, sintomas associados, duração da alteração do olfato, dia e forma de instalação, comorbidades, necessidade de internação e tratamento realizado.

Os dados obtidos alimentaram uma planilha criada no *Google Drive* com acesso aberto ao grupo de pesquisadores. Esses resultados são apresentados de forma descritiva demonstrando a prevalência desses eventos na população estudada.

## RESULTADOS

No período entre 4 de junho e 14 de julho de 2020, 13.910 pacientes com diagnóstico clínico ou laboratorial de COVID-19 foram telemonitorados pela Secretaria de Saúde e a UFG, sendo que, destes, 627 relataram alteração no olfato, resultando na prevalência de 4,51% de alteração de olfato nessa população.

Dos 627 pacientes com alteração de olfato que foram triados, 62% eram do sexo feminino e 38% do masculino. Obteve-se contato com 330 pacientes, os quais foram incluídos e concordaram em responder o questionário específico. Destes, 62% eram do sexo feminino ( Figura 1 ). Quanto à idade, 86% dos pacientes tinham menos que 50 anos; destes, 36% tinham menos que 30 anos. Em relação à ocupação profissional, cerca de 23% dos entrevistados eram profissionais de saúde. As entrevistas foram realizadas, em média, nos primeiros 10 dias da doença e, quanto ao diagnóstico, 64,2% dos pacientes já tinham confirmação por testes específicos.

**Figura 1:** Distribuição por sexo

F: feminino; M: masculino.

Avaliando o quadro clínico, o sintoma inicial apresentado mais prevalente foi cefaleia, por quase 30% dos pacientes, seguido por mialgia (23%) e febre (18%); a perda de olfato foi o sintoma inicial em 12% dos pacientes ( Figura 2 ). Sobre a quantificação subjetiva da perda olfatória, 81,3% relataram perda total do olfato (anosmia), enquanto 18,6% relataram perda parcial (hiposmia). A perda olfatória se instalou nos primeiros 5 dias em 80% dos pacientes, sendo que, na maioria, ocorreu de forma súbita (70%) ( Figura 3 ). A recuperação total do olfato até o décimo dia de doença foi observada em somente 13% dos pacientes, 38% tiveram melhora parcial e 48% se mantiveram com anosmia até o momento em que se realizou a entrevista. Os pacientes que não recuperaram totalmente o olfato continuaram em seguimento via telemonitoramento junto à equipe de otorrinolaringologia, com ligações periódicas em 15 e 30 dias, até a melhora do sintoma de anosmia.

**Figura 2:** Sintoma inicial

**Figura 3:** Dia de instalação da perda de olfato

Analisando os sintomas associados ( Figura 4 ), chamou atenção a disgeusia, presente em 89% dos pacientes, sugerindo forte relação com a anosmia/hiposmia. Cefaleia e mialgia seguiram com maior prevalência, aparecendo em 82% e 75% dos pacientes, respectivamente. A obstrução nasal foi relatada por 69% dos pacientes, rinorreia por 60%, tosse por 69%, disfonia por 42% e dispneia por 36%. A prevalência de obesidade foi 20%, sendo 14% com obesidade grau 1, 37% com sobrepeso e índice de massa corporal normal em 39% dos pacientes.

**Figura 4:** Sintomas associados

Sobre o tratamento realizado, 50% fizeram uso de analgésicos comuns, 40% tomaram azitromicina, 25% ivermectina, 12% corticoide e 5,5% usaram hidroxicloroquina. O tratamento foi realizado ambulatorialmente em 97,3% dos pacientes, e somente 2,7% dos pacientes necessitaram de internação hospitalar, sendo que, destes, 77% ficaram internados por menos de 5 dias em enfermaria. Dos 330 pacientes, somente 0,6% necessitou de internação em unidade de terapia intensiva (UTI).

Em relação ao histórico otorrinolaringológico, aproximadamente 20% dos pacientes relataram algum acometimento nasal, como rinite (10,3%), rinossinusite (7,8%) e obstrução nasal (2,1%). Outras comorbidades também foram questionadas, e 9,7% relataram hipertensão, 5,1% diabetes e 6,9% asma; nenhum paciente relatou insuficiência cardíaca.

Sobre a realização de exames complementares, 83% dos pacientes não fizeram nenhum exame laboratorial. O exame mais realizado foi o hemograma, por cerca de 17% dos pacientes, sendo que se encontrou normal em 73% dos casos, apresentou leucopenia em 7% e plaquetopenia em 17%. Dímero-D foi realizado somente em 1,9% dos pacientes e apresentou-se normal em todos eles. Radiografia de tórax foi feita em 5,8% dos pacientes e teve alteração em 0,9%; entretanto, 14,7% dos pacientes foram submetidos à tomografia de tórax; 47% desses exames estavam normais, 30% tinham menos de 25% de acometimento pulmonar, 12% menos de 50% de acometimento e somente 2% mais de 50% de acometimento pulmonar sugestivo de pneumonia viral.

## DISCUSSÃO

Segundo a literatura científica atual, a prevalência de anosmia relacionada à infecção pelo SARS-CoV-2 é muito variável (5% a 85,6%). Isso acontece provavelmente devido às diferentes metodologias utilizadas e pela inclusão de pacientes suspeitos.^( 1 , 6 )^Neste estudo, foi encontrada taxa de 4,51% nos pacientes telemonitorados pela Secretaria de Saúde e pela UFG, que é um número baixo, provavelmente pela presença de muitos pacientes suspeitos, sem diagnóstico confirmado por laboratório, indicando um viés de seleção desses pacientes. Quando analisados os dados dos pacientes selecionados para o questionário (n=330), encontram-se dados mais concordantes com a literatura, provavelmente porque todos esses pacientes tinham queixa de perda de olfato e, como esse sintoma parece ser preditor positivo da doença, essa seleção diminui os casos suspeitos que são verdadeiramente negativos.

Estudos recentes realizados em amostras populacionais em períodos que precederam a pandemia de COVID-19 apontam como fatores de risco para distúrbios do olfato o sexo masculino e o avanço da idade, com diferenças estatisticamente significativas entre os sexos e faixa etária entre 18 e 50 anos (mulheres apresentaram melhor função olfativa em testes subjetivos nessa faixa).^( 7 , 8 )^Em contrapartida, estudos descritivos realizados em populações com COVID-19 mostraram que as alterações de olfato predominam no sexo feminino, mas a média de idade dos pacientes analisados foi similar.^( 2 , 9 - 12 )^Observam-se então, neste estudo, semelhanças nas distribuições com os estudos anteriores, com 67,2% dos pacientes sendo mulheres, com média de idade de 37 anos.

Em relação à proporção de pacientes com diagnóstico confirmado por testes específicos para COVID-19 nesta amostra, 64,2% tinham a confirmação laboratorial no momento da entrevista, e os demais tinham quadro clínico sugestivo, mas ainda sem confirmação laboratorial. A análise da ocorrência de anosmia e hiposmia nesses dois grupos é convergente com um grande estudo multicêntrico realizado por Parma et al., em que, nos dois grupos estudados, não houve diferenças estatisticamente significativas em relação à prevalência dos distúrbios do olfato (apenas uma intensidade maior na redução do olfato no grupo com o diagnóstico laboratorial confirmado).^( 13 )^Foi feita a mesma análise com os presentes dados e confirmou-se que os pacientes com diagnóstico laboratorial tiveram taxa maior de anosmia (82%), e aqueles com diagnóstico clínico tiveram maior taxa de hiposmia (72%). Apesar disso, a taxa de ageusia nos dois grupos foi alta e similar (85% primeiro grupo e 93% no segundo grupo). Essa análise leva a crer que, apesar de cerca de 36% dos pacientes da coorte avaliada não terem teste de confirmação do diagnóstico de COVID-19, o quadro clínico presente em todos os pacientes entrevistados sugere boa equivalência e mantém a confiabilidade dos dados obtidos.

Quanto à diferenciação da intensidade do distúrbio do olfato referida nesta amostra (81,3% com anosmia e 18,6% com hiposmia), dados semelhantes são dispostos em estudo com metodologia semelhante (questionário *on-line* ), que apresentou como resultados a prevalência de 81,7% e 76,1% de anosmia, além de 15,1% e 17,9% de hiposmia, em pacientes em quarentena e com o diagnóstico confirmado laboratorialmente, respectivamente.^( 10 )^Hopkins et al., e Kosugi et al., também observaram padrão parecido de distribuição entre a intensidade da perda de olfato.^( 9 , 14 )^

O distúrbio do olfato foi o primeiro sintoma de COVID-19 em 11,8% dos casos no estudo de Lechien et al., semelhante aos 12% encontrados neste estudo; porém a taxa de apresentação como sintoma inicial varia muito em diferentes séries (de 3% a 83%).^( 2 , 11 , 14 - 22 )^Observa-se, nesta série, que a instalação da perda do olfato se deu de forma súbita em 70% dos casos, e ela ocorreu até no quinto dia de sintomas da doença em 80% dos entrevistados, semelhantemente ao observado nos estudos de Hopkins et al., e Klopfenstein et al.,^( 9 , 12 )^

Quanto à evolução, 48% dos pacientes persistiram com a alteração olfatória até o décimo dia de sintomas. Apesar da persistência da queixa, a fisiopatogenia do distúrbio olfatório na COVID-19 apresenta mecanismos não neuronais, o que sugere que a total perda olfativa permanente seria improvável.^( 23 )^

Em relação à associação de distúrbios do olfato com distúrbios da gustação, outros estudos semelhantes corroboram estes achados (89%), com variação entre 85% a 90,65%.^( 2 , 9 , 12 )^Já em relação à presença concomitante de sintomas nasais, nesta amostra foi de aproximadamente 20%, sendo menor do que o encontrado em outros estudos.^( 10 , 14 )^Quanto à prevalência de sintomas inespecíficos, como cefaleia (em 85% dos pacientes) e mialgia (em 75%), foi percebido, nesta série, perfil semelhante ao achado de estudo que testou o olfato objetivamente de pacientes confirmados laboratorialmente.^( 24 )^

Nesta análise, os pacientes apresentaram um tempo maior referido para a melhora do olfato, mesmo que parcial, em comparação a outros estudos.^( 10 )^Apesar disso, são similares com outra série brasileira, na qual também se discute um maior tempo de recuperação em pacientes com alterações do olfato devido à COVID-19 do que em outros quadros pós-virais.^( 14 )^

Os casos que necessitaram internação hospitalar ou cuidados de terapia intensiva foram menores do que aqueles citados por Klopfenstein et al., em estudo retrospectivo de pacientes que buscaram atendimento médico e tiveram diagnóstico confirmado de COVID-19.^( 12 )^Isso pode ser explicado pelo fato de que este estudo foi realizado por meio de inquérito feito por telefonema e, portanto, em pacientes que, em um primeiro momento, teriam quadros leves a moderados, mas não se pode ignorar a possível relação da presença de anosmia como fator protetor de gravidade, como já aventado por outros estudos.^( 2 )^De fato, faltam dados científicos sobre anosmia em pacientes com quadros moderados a graves, talvez pelo foco nesses casos ser no tratamento ou na investigação de outros fatores potencialmente mais graves, como dispneia. Médicos de unidade de terapia intensiva e pacientes com quadro grave podem não perceber ou não dar importância para a presença do sintoma anosmia diante do quadro sistêmico grave.

Este estudo possui diversas limitações, como a obtenção de dados a partir de avaliações subjetivas dos participantes e estando, assim, sujeito a vieses culturais e de aferição. Entretanto, obteve-se uma amostragem ampla de uma única região, e foi realizado a partir de contato direto de otorrinolaringologistas com os pacientes, permitindo caracterizar com detalhes o panorama dos distúrbios do olfato durante o período de maior crescimento de casos em Goiás.

Outra limitação é a falta de confirmação laboratorial de todos os casos, mas é importante lembrar que a presença de anosmia como sintoma isolado possui associação positiva com o diagnóstico de COVID-19 quando em comparação com outros vírus respiratórios, sendo essa associação ainda mais forte quando há ausência de outros sintomas nasais.^( 16 , 21 )^

Ressalta-se ainda que os testes laboratoriais possuem diferentes capacidades de detecção do SARS-CoV-2, a depender do dia da coleta, da técnica e do tipo de material coletado para a amostra, sendo que os *swabs* nasofaríngeo e orofaríngeo (técnicas coleta de amostras para RT-PCR mais utilizadas no meio) possuem apenas moderada capacidade de detecção, com consideráveis taxas de falso-negativos.^( 25 - 29 )^Dessa forma, reforça-se a importância das alterações olfativas na presunção do diagnóstico de COVID-19.

A hiposmia ou anosmia súbitas, suas associações com alteração do paladar e a ausência de outros sintomas nasais (como obstrução nasal e rinorreia) devem ser consideradas importante fator preditivo positivo do diagnóstico de COVID-19 e, uma vez que costumam surgir precocemente, configuram-se como importante ferramenta a fim de interromper a disseminação viral. No contexto pandêmico atual, concorda-se que a presença desse quadro clínico deve levantar suspeita imediata da doença, e sugerir quarentena para esses pacientes, antes mesmo do exame confirmatório.^( 30 - 32 )^Essa associação tem grande valor social, já que, no nosso país, ainda não é possível a testagem de todos os casos suspeitos, e, por se tratar de uma doença ainda pouco conhecida, acredita-se que os dados deste estudo podem colaborar com a literatura nacional e também mundial. Também reforça-se que deve haver suspeição de resultados falsos-negativos diante de caso clínico típico – fator importante, visto a baixa sensibilidade para fase aguda dos testes moleculares e sorológicos.^( 16 , 21 , 30 )^

Sobre a evolução da anosmia, é salutar o acompanhamento desses pacientes, para avaliar a evolução do quadro, uma vez que tais sintomas causam relevante impacto psicossocial.^( 6 )^São necessários mais estudos quanto a evolução a longo prazo da anosmia e fatores relacionados ao tratamento. Estudo recente de Brann et al., sugeriu o acometimento de células não neuronais como causa da anosmia na COVID-19, sendo provável a recuperação completa da maioria dos pacientes afetados.^( 23 )^O acompanhamento dos pacientes desta amostra cuja alteração do olfato persiste deve ser mantido mensalmente, até a resolução total.

Salienta-se que, apesar da relação da perda olfatória com casos leves de COVID-19, ainda não há, na literatura, estudos sobre esse sintoma em pacientes com quadros moderados e graves, de forma que essa relação pode ser um viés de aferição.^( 33 )^Por isso, sugerem-se à comunidade científica mais pesquisas sobre o assunto.

## CONCLUSÃO

Neste estudo, realizado via inquérito por telefonema, com pacientes com distúrbio do olfato que estavam em quarentena por quadros suspeitos ou confirmados laboratorialmente de COVID-19, foi encontrada uma incidência de perda de olfato maior no sexo feminino, sendo a instalação de forma súbita a mais comum. O sintoma associado à perda de olfato mais comum foi a perda de paladar, e a presença de ambas as queixas deve aumentar a suspeição do diagnóstico de COVID-19.

## Apêndice 1

**Table:** Apêndice 1. Questionário dos dados epidemiológicos

O questionário foi realizado via telefonema com recursos próprios de cada pesquisador. Após apresentação pessoal do entrevistador e do projeto de telemonitoramento, foi obtido um consentimento verbal do paciente para responder as seguintes perguntas:
- Sexo.
- Idade, peso, e altura.
- Data de nascimento.
- Profissão.
- Qual foi o primeiro sintoma?
- Há quantos dias teve o primeiro sintoma?
- Quando e qual teste confirmatório de COVID-19 foi realizado?
- Qual o dia da alteração de olfato?
- Classificar entre hiposmia e anosmia.
- Realizou algum teste de olfato?
- Como foi a instalação da perda de olfato (súbita/ progressiva)?
- Qual a duração da perda de olfato?
- Se teve melhora da perda de olfato ou persiste?
- Presença dos sintomas: alteração de paladar, espirros, dor de garganta, obstrução nasal, rinorreia, disfonia, tosse, dor facial, cefaleia, mialgia, dispneia, dor torácica, febre, dor abdominal, diarreia e náuseas?
- Qual tratamento está sendo realizado?
- Houve necessidade de internação?
- Histórico específico de doenças e/ou procedimentos otorrinolaringológicos.
- Presença de comorbidades e história prévia: diabetes, hipertensão, doença pulmonar, doença com comprometimento imunológico, insuficiência cardíaca, doenças cardíacas que não insuficiência cardíaca, câncer, e outras doença não especificada previamente.
- Resultados de exames complementares: laboratoriais e tomografia de tórax.
- Observação para outros detalhes não abordados nas perguntas anteriores.
Os pacientes que não tiveram melhora total da anosmia/hiposmia serão contatados novamente, 15 e 30 dias após a primeira ligação, para questionamento da melhora dos sintomas olfatórios e gustatórios. Após 30 dias, caso persista com perda de olfato, o paciente é convidado a uma avaliação presencial ambulatorial e à aplicação de teste de olfato.

COVID-19: doença pelo coronavírus 2019.
